# How Does the Frequency of Eating-Alone among Older People in Korea Affect Their Health and Dietary Behavior?

**DOI:** 10.3390/nu15092085

**Published:** 2023-04-26

**Authors:** Yongseok Kwon, Kyung Hee Hong, Yoo-Kyung Park, Sohye Kim

**Affiliations:** 1 National Institute of Agricultural Sciences, 166 Nongsaengmyeong-ro, Wanju 55365, Republic of Korea; 2 Department of Food Science and Nutrition, Dongseo University, Busan 47011, Republic of Korea; 3 Department of Medical Nutrition, Graduate School of East-West Medical Science, Kyung Hee University, Yongin 17104, Republic of Korea; 4 Nutrition Care Services, Seoul National University of Bundang Hospital, Seongnam 13620, Republic of Korea

**Keywords:** eating alone, mental health, old people, Korea National Health and Nutrition Examination Survey (KHANES)

## Abstract

This study examined the relationship between general population characteristics and diet-related factors pertaining to eating alone for older adults (65 years and older) in Korea. This study used the Korea National Health and Nutrition Examination Survey (KNHANES), 2016–2020, and the target population was 7037 Koreans aged 65 years or older who participated in the nutritional survey and health interview. Eating alone variables were classified as follows. Eating together all day means “eating together”, eating only one meal a day means “1/day”, eating two meals a day alone means “2/day”, and “3/day” means eating three meals a day alone. The main results are as follows. The rate of moderate or severe food insecurity was 3.41% in the “eating together” group to 7.86% in the “3/day” group, which was 4.45% higher in the “3/day” group. Fruit + vegetable intake among food intake lowered by about 35 g from 301.2 g in the “eating together” group to 266.2 g in the “3 day” group. In addition, as a result of analyzing the prevalence of depression using the PHQ-9 score, the “3/day” group had a 1.775 to 2.464 times higher risk of depression than the “eating together” group. Finally, EQ-5D variables and quality of life scores were significantly lowered from the “eating together” group to the “3/day” group. Overall, higher frequency of eating alone was associated with food safety, essential food intake, and quality of life. Based on these results, it is thought that a dietary life support program such as the eating together program is necessary to improve the quality of life of the older people who eat alone.

## 1. Introduction

The number of people who eat alone is increasing in Korea. In the past, Korean culture was characterized as collective, with eating together as a social norm. The eating-alone habit has emerged as a typical eating pattern in modern Korean society and even a new acronym, “Hon-Bab”, has been created to describe it [1,2,3,4]. The increase in the age of first marriage and the prevalence of single-person households due to the aging population due to a change in the population’s age structure are the main reasons for the increase in eating alone. This change can be seen as a cultural phenomenon of a modernized society emphasizing efficiency, even in how effectively people use break time, or as a social structure change toward individualization [5,6,7,8]. According to the Statistics Korea report, single-person households comprised 20.0% of the entire population in 2005, 27.2% in 2015, and 32.4% in 2021, rapidly increasing over the last 16 years [9]; it mentioned that eating alone occurs in single-person households or among people in their 20s and 30s. However, eating alone now is becoming more common in people of all ages and generations, not just the younger generation [3,4,10]. The disadvantages of eating alone include a lack of intake of various nutrients and food groups, as fast foods (e.g., ramen, bread, gimbap, and sandwiches) are generally preferred [11,12,13]. According to previous research, eating alone leads to insufficient protein and calcium intake and is associated with an excess intake of carbohydrates and sodium, leading to nutritional imbalance. The risks associated with eating alone for obesity, hypertension, and metabolic syndrome are high, increasing concerns over eating habits, health, and nutrition management [14,15,16].

It has been reported to cause adverse effects on diet quality and eating habits, particularly in older people, due to the high frequency of eating alone, low dietary diversity, high meal-skipping rates, and inadequate consumption of various nutrients and food categories [17,18,19,20,21,22]. It was also found that cognitive function was reduced significantly and nutritional status was poorer for the older adults who ate alone compared to those who ate together; eating alone was a significant risk factor for high levels of social isolation and depression symptoms. [17,20,23,24,25,26,27,28]. Additionally, eating alone was associated with higher mortality risk and feeling unhappy, resulting in lower quality of life and the increased likelihood of multiple comorbidities [17,21,29,30,31]. Based on the results of previous studies reporting the risk factors associated with eating alone, it seems to affect both physical and mental health negatively.

Previous studies about eating alone conducted in Korea did not focus on older adults. For example, the topics of these studies were “the comparison of perceptions about eating alone and eating behaviors between male and female university students”, “the relationship between nutrient intake and metabolic syndrome for middle-aged adults who eat alone”, “comparison between eating with family and eating alone in adolescents”, and “the effects of eating alone or eating together with the family on pediatric mental and physical health.” Additionally, a study on older individuals that investigated the association between eating alone and depression in older Korean females did not examine the frequency of eating alone as an eating behavior in relation to health-related indices [12,32,33,34,35,36]. Thus, the purpose of this study is to determine the frequency of eating alone among older adults aged 65 years or older [12,32,33,34,35,36]. In addition, we aim to describe the general characteristics of this population and then identify individual eating habits. Further, we intend to learn how food and nutrient intake affect depression and quality of life. We hope that the results of this study, obtained by identifying the relationship between the frequency of eating alone as an eating pattern and mental health risk factors, will help older adults maintain an appropriate diet and manage their health status in preparation for the hyper-aging population. 

## 2. Materials and Methods

### 2.1. Research Data

This study was a sub-study of the Korea National Health and Nutrition Examination Survey (KNHANES) conducted in 2016 and 2020. The KNHANES administered by the Korean Ministry of Health and Welfare and a stratified multistage probability design were used with subject selection from sampling units and using household registries. KNHANES is a nationwide, population-based, cross-sectional study aimed at the health and nutrition status of the noninstitutionalized civilian Korean population. In addition, it provides basic data for health policies such as improvement of people’s nutrition, disease prevention, and development of health promotion programs [37]. The KNHANES consists of health interviews, health examinations, and nutritional surveys, among which the nutritional survey aims to understand the food and nutritional intake and dietary habits of Koreans.

In this study, a 24-h recall method was used as a nutrition survey. Food and nutrient intake and dietary behavior were investigated and smoking, alcohol consumption, exercise, mental health, and life quality were examined in a health survey [37]. 

### 2.2. Subjects

**Figure 1:**
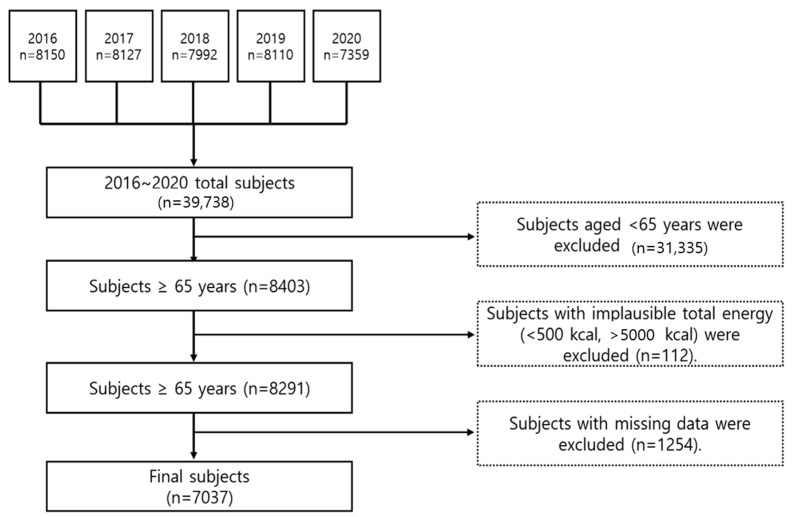
The flowchart for subject samples of this study.

This study selected older people over 65 years old who participated in the nutritional survey and health interview of the KNHANES 2016–2020. Some of the subjects who consumed less than 500 kcal or more than 5000 kcal (112 people) were excluded. Outlier data on subjects who did not participate in the dietary survey (24-h recall survey) were also excluded (1254 people). A total of 7037 people were selected in the study (Figure 1). The KNHANES data used in this study were approved by the KCDA (Korea Disease Control and Prevention Agency) Institutional Review Board (IRB approval numbers: 2018-01-03-P-A, 2018-01-03-C-A, and 2018-01-03-2C-A). Among these, the 2016 and 2017 KNHANES were exempt from review regarding research ethics based on the Bioethics and Safety Act from 2015 to 2017 [38].

### 2.3. Meal Pattern According to the Frequency of Eating Alone

In this study, we used the variables recorded in the “24-h recall to measure the eating alone variable.” First, respondents who consumed more than two daily meals were targeted. The variables “meals with other people” (variable name: N_meal_w) and “number of meals” (variable name: N_meal) were used. “Eating together group” consisted of people who had all daily meals—namely breakfast, lunch, and dinner—with their family members or others throughout the day; “1/day eating alone group” for those who ate alone only once a day; “2/day eating alone group” for those who ate alone twice a day, and “3/day eating alone group” for those who ate alone thrice a day. 

### 2.4. General Characteristics

General characteristics of the subjects, such as gender, age, residential area, marital status, household number, education level, household income, and job status were analyzed. Among these characteristics, marital status was classified as single or married, residential area was classified as urban or rural area, and household number was classified as “1 person”, “2 people”, “3 people”, “4 people”, “5 people” and “over 6 people”. The education levels were classified as “Middle school or less”, “High school or less” and “College or more”. Household income level was classified using the household income level variables in KNHANES and job status was classified as employed or unemployed.

### 2.5. Health Related Characteristics

For health behavior, smoking, drinking, stress, exercise, and obesity status were analyzed. Obese status was classified as underweight (BMI < 18.5 kg/m^2^), normal (18.5 kg/m^2^ ≤ BMI < 23.0 kg/m^2^), overweight (23.0 kg/m^2^ ≤ BMI < 25.0 kg/m^2^), and obese (BMI ≥ 25 kg/m^2^). 

### 2.6. Dietary Behavior

Dietary behaviors were analyzed for snack consumption, food security, and eating-out frequency. For snack intake, using the meal-related variables in the 24-h recall data, those who chose snacks were classified as “yes” and those who did not were classified as “no”. Food security was determined by using variables of dietary life and classified by referring to previous studies [39,40].

### 2.7. Food and Nutrient Intake

Food intake was categorized into 17 food groups using food classification codes and food intake in the food (24-h recall) data; individual intake was obtained for each survey subject. In addition, energy, carbohydrate, protein, fat, Ca, P, Fe, Na, K, vitamin A, carotene, retinol, thiamine, riboflavin, niacin, and vitamin C intake and contribution ratios were calculated using the daily intake for each nutrient.

### 2.8. PHQ-9

The Patient Health Questionnaire-9 (PHQ-9) was used in the National Health and Nutrition Examination Survey to measure depression and the questionnaire consisted of nine items. For each question, subjects were instructed to choose one of: “none” (0 points), “several days” (1 point)”, “more than a week” (2 points), or “almost every day” (3 points) and score all of the corresponding questions. The total score was calculated by adding them up. A higher PHQ-9 score means more depressive symptoms.

### 2.9. EQ-5D

Quality of life was measured using the Euro Quality of Life five-dimension (EQ-5D) tool. EQ-5D is a five-item measurement tool that can be easily used to determine the level of physical health, mental health, and social health. The items are composed of motor ability, self-management, daily activities, pain/inconvenience, and anxiety/depression and are evaluated as “no problem”, “some problem”, and “severe problem”, respectively. The score is calculated by applying a weight to each item value and the value range is 162 between −1 point and 1 point, and the lower the score, the lower the quality of life [36].

### 2.10. Statistical Analysis

The KNHANES data was obtained by stratified multi-stage sampling rather than a simple random sampling method. The collected data were analyzed with a consideration of weight, strata variable (KSTRATA), and cluster variable (Primary Sampling Unit, PSU). Among them, general matters, dietary factors, and categorical variables such as health-related factors, frequency analysis (frequency analysis) was performed to determine the frequency (n) and the weighted percentage (weighted %) and the significance test was a chi-square test. For qualitative variables, descriptive analysis was performed and the mean and standard error were expressed. For the significance test of these continuous variables, the p for trend value was obtained using the SURVEYREG procedure. Among them, in the case of nutrients and food intake, the adjusted p for trend value was obtained by correcting for age and energy intake. In addition, multiple logistic regression analysis was conducted to analyze the relationship between the prevalence of eating alone and the prevalence of depression. Multiple regression analysis was conducted to determine whether the prevalence of eating alone was related to quality of life. All statistical analyzes were performed using the SAS ver. 9.4 package (SAS Institute, Cary, NC, USA), and the significance level was set at α = 0.05.

## 3. Results 

### 3.1. General Characteristics

Table 1 presents the general characteristics of the target population. According to the results of the 24-h recall survey, the proportion of males was higher than that of females in the “eating together” group, while the proportions of females were higher in the “1/day eating alone”, “2/day eating alone”, and “3/day eating alone” groups (*p* < 0.0001). The proportion of females and the mean age increased in proportion to the frequency of eating alone. The mean ages for the “eating together” group and the “3/day eating alone” group were 72.82 and 74.19 years old, respectively (*p* < 0.0001). Regarding the area of residence, more subjects in the “3/day eating alone” group lived in “urban” rather than “rural” areas, compared to those in the “eating together” group (*p* < 0.0001). The “household number” of subjects, which is the proportion of families consisting of two members, was the highest in the “eating together” and “1/day eating alone” groups. In contrast, the proportions of the families consisting of one and two members were similar to each other in the “2/day eating alone” group and that of one member was the highest (55.06%) in the “2/day eating alone” group (*p* < 0.0001). Regarding education level, “middle school or less” was the highest and the higher the frequency of eating alone, the lower the level of education. As for household income, the proportion of “low” was high in the “3/day eating alone” group and for “job status”, the proportion of “3/day eating alone” was higher in the “unemployed” group than in the “employed” group (*p* < 0.05).

### 3.2. Meal Pattern According to the Frequency of Eating Alone

Table 2 shows meal patterns according to the frequency of eating alone. Significant differences were found in all results (*p* < 0.001). Of all subjects aged 65 or older, 42.37% ate all three meals daily with other people. The higher the frequency of eating alone, the lower the proportion of subjects: 23.58% for “1/day eating alone”, 20.10% for “2/day eating alone”, and 13.95% for “3/day eating alone.” Most in the “eating together group” were found to eat with “family” and “family and other people.” In the “1/day eating alone” group, the “family + alone” rate was the highest; in the “2/day eating alone” group, “only alone” was 24.92%, “family + alone” was 39.31%, and “other people + alone” was 35.77%. The results from analyzing eating alone during the day in the “1/day eating alone” group “Breakfast (B)” was 40.28%, “Lunch (L)” was 38.99%, and “Dinner (D)” was 20.73%, demonstrating that eating alone was higher for breakfast and lunch than dinner. In the “2/day eating alone” group, the “B + D” rate was the highest (47.23%), followed by “B + L” (33.30%), and “L + D” was the least (19.47%), showing that eating alone was the most frequent for breakfast. For “Cooking place for a solo meal”, for all of the “1/day eating alone”, “2/day eating alone”, and “3/day eating alone” groups, “only home (H)” was the most frequent, followed by “home + commercial (H + C)”, showing that older adults tend to prepare food at home to eat alone. 

### 3.3. Health-Related Characteristics According to the Frequency of Eating Alone

Table 3 presents the subjects’ health-related factors. The proportion of “non-smokers” was the highest in all groups (*p* < 0.0001), whereas drinking “<1/year” had the highest proportion (*p* < 0.0001). Additionally, the higher the frequency of eating alone, the lower the frequency of drinking. For stress, the subjects who reported a relatively higher number of meals eaten alone were likely to answer “feel it very much” (*p* < 0.0001). However, there was no significant difference between the exercise group and the obese group. 

### 3.4. Dietary Behavior According to the Frequency of Eating Alone

Table 4 presents the subjects’ dietary behavior based on the frequency of eating alone. Snack intake rate was lower when eating alone more frequently (*p* < 0.01). In the “food security” variable, the ratio of “Enough food—secure” decreased as the frequency of eating alone increased. In the “eating together” and “1/day eating alone” groups, the proportion of “enough food—secure” was high, measured by “all of our family could eat enough food and a variety of foods as much as they wanted.” In contrast, in the “2/day eating alone” and “3/day eating alone” groups, the mild food insecurity rate was high, measured by “all of our family could eat enough food but could not eat various kinds of food.” Regarding eating out, a higher frequency of eating alone was associated with eating out more frequently. The “3/day eating alone” group showed the highest proportion of rarely eating out (*p* < 0.0001).

### 3.5. Food Intake in Their Meal According to Consumption Frequency of Eating Alone

Table 5 presents the results from analyzing the subject’s food intake depending on the frequency of eating alone. The results show that the higher the frequency of eating alone, the lower the intake of “total food”, “Fruit”, “Meat, poultry, and their products”, and “Fish and shellfish”, and the greater the intake of “cereal and grain products” and “legumes and their products.” When adjusted for age, gender, and energy intake, the intake of “total food”, “vegetables”, “fruit”, “meat, poultry, and their products”, “seaweed”, and “seasonings” was significantly reduced (adjusted *p* for trend < 0.05).

### 3.6. Nutrient Intake Depending on the Consumption Frequency of Eating Alone

Table 6 presents the results of analyzing the subjects’ nutrient intake. After adjusting for gender, age, and energy intake (adjusted *p* for trend < 0.0001), the higher the frequency of eating alone, the lower the intake of protein, sodium, potassium, vitamin A, carotene, and niacin. Additionally, the rate of carbohydrate intake increased as the number of people eating alone increased, while the intake of protein and fat was significantly reduced.

### 3.7. Depression-Related Survey Results

Table 7 presents the results of evaluating the subject’s depression using PHQ-9. The total scores for the “3/day eating alone”, “2/day eating alone”, “1/day eating alone”, and “eating together” groups were 2.40, 2.06, 1.80, and 1.56, respectively, indicating that the more frequently people ate alone, the more depressed they felt. Looking at the prevalence of depression against the frequency of eating alone, the numbers for the “3/day eating alone”, “2/day eating alone”, “1/day eating alone”, and “eating together” groups were 6.16%, 3.89%, 3.35%, and 2.70%, respectively, indicating that a higher frequency of eating alone was linked to a higher prevalence of depression. 

### 3.8. Association between the Prevalence of Depression and the Frequency of Eating Alone 

The results of the logistic regression analysis to identify the relationship between the frequency of eating alone and depression are provided in Table 8. Using the “eating together” group as a reference, the unadjusted Model 1 revealed a trend of increasing prevalence of depression as the frequency of eating alone increased (*p* for trend d < 0.0001). Model 2, adjusted for gender, age, and energy intake, showed a significant increase in the prevalence of depression regardless of gender, age, or energy intake (*p* for trend < 0.01). However, statistical significance did not exist in Model 3, which was adjusted additionally for exercise, alcohol consumption, stress status, smoking, and obesity status. Model 4 was adjusted for food security, snacking, the frequency of eating out in a week, the frequency of having breakfast in a week, marital status, occupation, education level, and household income.

### 3.9. Quality of Life Survey Results

Table 9 shows the results of using EQ-5D to examine the subjects’ quality of life. The quality of life in the “3/day eating alone” group was significantly lower than the “eating together”, “1/day eating alone”, and “2/day eating alone” groups in all of the “mobility” (*p* < 0.001), “self-management” (*p* < 0.05), “daily activities” (*p* < 0.0001), “pain/inconvenience” (*p* < 0.05), and “anxiety/depression” (*p* < 0.01) items. 

### 3.10. Relationship between the Frequency of Eating Alone and Quality of Life

Table 10 presents the results of the relationship between the frequency of eating alone and quality of life. Using the “eating together” group as a reference, the unadjusted Model 1 showed significantly decreasing quality of life as the frequency of eating alone increased. In Models 2 and 3, with stepwise adjustment for age, gender, energy intake, family size, exercise, alcohol consumption, stress status, smoking, obesity status, food security, snacking, and the frequency of eating out per week, quality of life tended to decrease as the frequency of eating alone increased (*p* for trend < 0.05). In contrast, no significant difference was found in Model 4, which was adjusted additionally for food security, snacking, eating out frequency per week, marital status, occupation, education level, and household income. 

## 4. Discussion

The “eating alone” population is increasing due to the increase in single-person households and other relevant societal changes, including individualization. Additionally, such a phenomenon is not merely due to personal preferences but also reflects social, structural, and cultural changes [24]. Several previous studies have reported that eating alone negatively influences health for various reasons without clarifying the reasons behind its increase [18,24,41,42,43]. Reports suggest that a lack of diverse nutrient and food intake should be associated with increased levels of social isolation, depression, and decreased quality of life [24,44]. This study identified 7037 older adults from the 2016–2020 KNHANES database and examined their health, diet, food and nutrient intakes, depression, and quality of life that depended on the frequency of eating alone. 

Previous studies about eating alone using the KNHANES dataset defined eating alone based on respondents answering “no” to the dietary life survey question about eating meals (i.e., breakfast, lunch, and dinner) together with family members and other people. In contrast, this study analyzed more objectively the frequency of eating alone and the presence or absence of eating together using the 24-h dietary recall method. We found a gender difference in the “eating alone” condition. The proportion of men was higher than that of women only in the “eating together” group, whereas the proportion of women was higher in all other “eating alone” groups. In a previous study on Korean adults aged 19 years or older, the male ratio was higher than that of females in the “eating together” and “1/day eating alone” groups. Compared to the higher proportion of women in the other “eating alone” groups, i.e., eating alone for two or three meals per day, we found that the frequency of eating alone was higher among women than among men in older adults. The average ages of subjects in the “eating together” and “eating alone” groups were 72.82 and 74.19 years, respectively. Thus, the higher the frequency of eating alone, the higher the age. In research involving adults and eating alone, the average age of those who ate alone for one meal per day and three meals per day was 45.3 and 54.7 years, respectively [44]. Previous studies reported that 7% or 12.2% of Korean adults eat three meals alone a day, whereas our study targeted older adults and found that those eating all three meals alone per day (3/day eating alone) were as high as 13.95%, higher than the adult group [45,46]. We also analyzed the data after classifying the subjects into “65–74 years old” and “≥75 years old” groups. The rate at which the frequency of eating alone increased gradually decreased in the “65–74 years of age” group, while for the “≥75 years old or older” group, “eating alone once a day”, “twice a day”, and ”thrice a day” were 35.07%, 43.69%, and 50.3%, respectively. Thus, the older respondents were, the more often they ate a daily meal alone. 

The results of analyzing the frequency of eating alone (a solo daily meal) among subjects in this study show that for those who have one meal alone per day, the solo meal was breakfast, lunch, or dinner 40.28%, 38.99%, and 20.73% of the time, respectively. Thus, the rate of eating alone is higher for breakfast or lunch than for dinner, which is consistent with the results of a study with Korean adults [46]. Additionally, most subjects answered “two” regarding family size. Older adults were more likely to eat together with family members than other people who were not family members. The highest rate of eating all three meals alone daily was 55.06% for those with a single-member family. This indicates that older adults eating alone are more significantly affected by the number of family members. For older adults who live alone, the frequency of eating alone will likely increase, leaving them deficient in healthy eating and nutrient consumption. Thus, interventions must be taken to prevent imbalanced meals and maintain correct dietary habits. 

In the “cooking place of solo meal” survey item for the subjects of this study, the ratio of “only at home” was 65.82% in all eating alone groups regardless of the frequency of eating alone, meaning that most of the solo meals were prepared at home, which is higher than that for adults. Thus, providing services such as nutrition management that focus on homemade, balanced and healthy meals may be beneficial for older adults who eat alone.

Looking at the subject’s “food security”, the rates of “enough food security” (i.e., all members within the family could eat a sufficient quantity and variety of foods) were higher in the “eating together” and “1/day eating alone” groups. In contrast, the proportion of “mild food insecurity” (i.e., everyone in the family ate enough food, but not a good variety) was high in the “eating alone two meals a day” and “eating alone three meals” groups. This indicates that the higher the frequency of eating alone, the lower the food security. This can be interpreted to mean that the frequency of eating alone is higher in lower-income households. 

Furthermore, the more frequently people eat alone, the less frequently they eat out. Snack intake also decreased as the frequency of eating alone increased. Thus, the number of meals eaten alone was related to total food intake, food safety, snack intake, and the frequency of eating out. 

Looking at the consumption of food groups according to the frequency of eating alone, food groups such as vegetables, fruit, meat, poultry, seaweed, and seasoning showed a significantly lower intake as the frequency of eating alone increased (*p* for trend < 0.05). The total intake of “fruit” and “vegetables” in the “eating together” group was 301.2 g, while it was 304.2 g, 250.1 g, and 266.2 g in the “eating alone” groups, i.e., one meal a day, two meals a day, and three meals a day, respectively. Thus, in all groups, the consumption was less than 400 g, which the World Health Organization (WHO) and the World Cancer Research Fund (WCRF) recommend. This is consistent with the results from previous studies on middle-aged adults eating alone that found the frequency of eating alone increased as the frequency of vegetable and fruit intake decreased. It is also consistent with the results from studies on older Japanese adults eating alone that found the consumption of green and yellow vegetables and fruits was lower in the older adults eating alone than that in older adults eating together with others [18,19,20,32,45]. This means that people who eat alone consume relatively fewer vegetables and fruit and overall fruit and vegetable intake was low for older adults in all groups. In particular, the consumption of fresh fruit and vegetables was insufficient to a greater degree in older adults who ate alone more frequently, indicating that this population needs a varied diet that includes fresh fruit and vegetables, new recipes, and the development of food products, and education emphasizing the intake of fruit and vegetables to maintain a healthy and balanced life [46]. 

Food groups were affected by the frequency of eating alone. The intake of “meat, poultry, and their products” was significantly lower as the frequency of eating alone increased. A previous study of Korean adults eating alone also reported a lower intake of the same food group. In a study of middle-aged men, the more men ate alone, the less calcium and protein-based energy they consumed, while their fat intake increased. Therefore, “meat, poultry, and their products” have become a common food group, of which both adults and older people ate less when they ate alone more frequently [32,47]. This explains the result of significantly reduced protein intake concurrent with gradually increasing carbohydrate intake for an increasing number of people eating alone. Our study targeted older adults and showed that “meat, poultry, and their products” intake was significantly reduced, pointing towards lower protein intake. Thus, we learned that protein intake could be lowered as the number of people eating alone increases, regardless of age. 

We examined the relationship between the number of people eating alone and the stress subjects felt. The higher the frequency of eating alone, the more significant the proportion of “feel it very much” responses. This indicates that a high frequency of eating alone might be related to older adult stress. Examining the connection between depression and the frequency of eating alone in models adjusted for gender, age, and energy intake, we found that depression was significantly higher in the “eating three meals a day alone” group. No significant difference was found when the model was adjusted for other variables. However, previous studies investigating depression revealed a relationship between dinner accompanied by other people and depression in Korean adults. Other studies reported that the greater the number of solo meals, the higher the rate of depression. Notably in a previous study on older adults, eating alone was reported as a significant risk factor for high PHQ-9 scores, indicative of depression [30,44,48]. This result is consistent with a previous study using the geriatric depression scale (GDS) in the elderly over 65 years of age, which indicated that older adults who ate alone had higher rates of depression than those who ate with others [46]. 

The frequency of eating alone differed in terms of the areas of residence. In the case of urban residents, it was shown to be gradually increasing along with the frequency of eating alone, consisting of 73.51%, 81.19%, 80.9%, and 78.75% in the “eating together”, “eating alone one meal a day”, “eating alone two meals a day”, and “eating alone three meals a day” groups. Conversely, rural residents reported a slower decline in the frequency of eating alone than urban residents.

Rural residents are less likely to eat alone than urban dwellers. Additionally, they frequently engage in social support or activities more closely tied to women’s organizations and village halls, enhancing their sense of community. These might help them mitigate the feeling of loneliness and lower their level of depression. Thus, encouraging community activities for healthier and more regular meals as well as providing community kitchens for older adults residing in urban areas who frequently eat alone—the population of lone eaters would be large in urban areas—may assist in securing mutual exchange, interaction, and food security among older adults, ultimately improving their mental health and quality of life [49,50]. Evaluating older adults’ quality of life in relation to eating alone extends the concept of health to cover the disease-free state, including overall well-being, social well-being, and mental health. This study also investigated quality of life using the EQ-5D score to find that the higher the frequency of eating alone, the lower the quality of life. We also found a significant relationship between older adults’ eating alone habits and quality of life scores, confirming its close relationship with various health variables [30]. 

This study has several limitations, which are described as follows. First, it is a cross-sectional study using the KNHANES dataset, so a causal relationship between eating behaviors depending on the frequency of eating alone, nutrient intake, and mental health could not be identified. Additionally, there was no information on the reasons for eating alone, i.e., whether it was voluntary or involuntary; information on eating snacks, whether alone or with others, was absent. The characteristics of eating habits, including the number of meals, meal time, and whether the meal was cooked or was an instant food, were not reflected in this study. Furthermore, an individual’s routine diet might not have been considered because intake status was analyzed using 24-h recall data. Additionally, there are multifactorial causes of loneliness and depression among older people, including family environment, presence or absence of a spouse, subjective health status, contact with neighbors and friends, living conditions, living environments, sociocultural perspectives, and other factors. A limitation of this study is that it did not include all of these factors.

Despite these limitations, this study targeted older adults aged 65 or more from the KNHANES dataset and conducted weighted data analysis to secure the sample’s representativeness for older Korean adults.

This study identified the relationship between dietary behavior, food and nutrient intake, and mental health in older adults eating alone, reflecting current social and cultural conditions. Previous studies defined eating alone as answering “no” to a dietary life survey question about eating together with family members or others based on meals (i.e., breakfast, lunch, and dinner). In contrast, this study is advantageous because we used objective data based on the 24-h dietary recall method to categorize the frequency of eating alone and analyze the prevalence of eating together. Therefore, eating alone is not a mere act of eating; it is closely related to one’s health status and influences mental health and quality of life. Thus, older adults who often eat alone deserve attention not only in terms of meal improvement, but also in terms of social, cultural, and social welfare perspectives—developing appropriate health policies and nutrition education programs to promote dietary life management and mental health. Our findings in this study could be used as substantial evidence for establishing guidelines for dietary management among older adults who frequently eat alone.

## 5. Conclusions

Among older adults who frequently eat alone throughout the day, the intake of “vegetables”, “fruit”, “meat, poultry, and their products”, “seaweed”, “milk and dairy products”, and “seasonings” was reduced, exhibiting low nutritional intakes of protein, sodium, potassium, vitamin A, carotene, and niacin. Although the food groups and single nutrient intakes used in this study could not determine the overall composition of the diet, they showed how meal patterns might differ depending on how often one eats alone. Thus, the results from our study could be useful for appropriate nutrition management and meal planning for older adults who frequently eat alone. Additionally, our results show that a higher frequency of eating alone influences depression and quality of life, indicating a need for future welfare services customized for older adults that could improve their health status.

Older adults who eat alone have different characteristics from age groups, so studies about eating alone improve our understanding of its frequency and the patterns of the meals eaten alone based on different genders and regions. The results from such studies could help establish suitable strategies for adequate nutrition and health management among older adults who frequently eat alone. 

## Figures and Tables

**Table 1:** General characteristics according to the frequency of eating alone.

Variables	Eating Together *n* = 3060 (42.37%)		Eating Alone						*p*-Value ^(2)^
			1/Day *n* = 1574 (23.58%)		2/Day *n* = 1394 (20.10%)		3/Day ^(3)^ *n* = 1009 (13.95%)		
	*n*	% ^(1)^	*n*	%	*n*	% ^(1)^	*n*	%	
Gender									<0.0001
Male	1519	52.03	686	45.48	473	36.50	357	38.53	
Female	1541	47.97	888	54.52	921	63.50	652	61.47	
Age									<0.0001
65–74	1844	60.54	1006	64.92	757	56.30	476	49.70	
≥75	1216	39.46	568	35.07	637	43.69	533	50.30	
Average (Mean, SE)	72.82 ^a^	0.12	72.32 ^b^	0.16	73.28 ^a^	0.16	74.19 ^c^	0.18	<0.0001 ^(3)^
Region									<0.0001
Urban	2075	73.51	1206	81.19	1046	80.90	740	78.75	
Rural area	985	26.49	368	18.81	348	19.10	269	21.25	
Marital status									<0.0001
Married	3035	99.80	1568	99.77	1373	98.46	995	98.78	
Single	5	0.2	6	0.23	21	1.54	14	1.22	
Household number									<0.0001
1	112	2.85	255	11.92	656	36.44	662	55.06	
2	2145	65.10	871	51.63	477	36.18	214	25.30	
3	476	18.26	251	20.08	150	16.12	73	11.22	
4	155	6.43	98	7.79	53	5.72	28	3.97	
5	103	4.16	62	5.95	40	3.93	22	3.47	
≥6	69	3.19	37	2.64	18	1.62	10	0.98	
Education level									<0.0001
Middle school or less	1925	64.60	953	63.23	917	69.45	702	75.93	
High school or less	561	20.98	289	21.71	216	18.92	136	16.88	
College or more	358	14.42	190	15.05	134	11.63	62	7.18	
Household income									<0.0001
Low	1306	40.99	603	36.79	744	50.58	665	60.10	
Middle-low	933	30.26	494	29.99	339	23.39	195	23.21	
Middle-high	492	17.11	280	19.26	180	15.69	100	11.32	
High	319	11.63	187	13.95	122	10.33	42	5.37	
Job status									0.0274
Employed	1018	33.47	521	36.15	384	30.43	273	30.32	
Unemployed	1829	66.52	911	63.85	883	69.57	629	69.68	

^(1)^ Weighted %. ^(2)^ *p*-value by chi-square test. ^(3)^ *p*-value by one-way ANOVA. ^a–c^ Different superscript letters mean “significantly different” among groups at α = 0.05 level by Tukey’s multiple range comparison.

**Table 2:** Consumption pattern according to the frequency of eating alone.

Variables	Eating Together *n* = 3060		Eating Alone					
			1/Day *n* = 1574		2/Day *n* = 1394		3/Day *n* = 1009	
	*n*	% ^(1)^	*n*	%	*n*	%	*n*	%
Eating partner among daily meal								
Everything-alone	-	-	-	-	349	24.92	1009	100.00
Family	2251	73.77	-	-	-	-	-	-
Family + alone	-	-	982	62.9	514	39.31	-	-
Other people	69	2.19	-	-	-	-	-	-
Other people + alone	-	-	289	17.7	531	35.77	-	-
Family + other people	740	24.0	-	-	-	-	-	-
Family + other people + alone	-	-	303	19.37	-	-	-	-
Alone meal in daily meal								
Breakfast ^(2)^	-	-	628	40.28	-	-	-	-
Lunch ^(3)^	-	-	611	38.99	-	-	-	-
Dinner	-	-	335	20.73	-	-	-	-
B ^(3)^ + L ^(4)^	-	-	-	-	431	33.30	-	-
B + D ^(4)^	-	-	-	-	628	47.23	-	-
L + D	-	-	-	-	262	19.47	-	-
B + L + D	-	-	-	-	-	-	1009	100.00
Cooking place of solo meal								
Only home	-	-	1190	74.30	979	69.06	690	65.82
Only commercial	-	-	374	25.18	85	5.98	23	2.01
Only institution	-	-	10	0.53	3	0.24	5	0.48
H ^(5)^ + C ^(6)^	-	-	-	-	318	24.05	282	30.86
H + I ^(7)^	-	-	-	-	6	0.34	3	0.23
C + I	-	-	-	-	3	0.33	3	0.30
H + C + I	-	-	-	-	-	-	3	0.29

^(1)^ Weighted %, ^(2)^ B: Breakfast, ^(3)^ L: Lunch, ^(4)^ D: Dinner, ^(5)^ H: Home, ^(6)^ C: Commercial place, ^(7)^ I: Institution.

**Table 3:** Health-related characteristics according to consumption frequency of eating alone.

Variables	Eating Together *n* = 3060		Eating Alone						*p*-Value ^(2)^
			1/Day *n* = 1574		2/Day *n* = 1394		3/Day *n* = 1009		
	*n*	% ^(1)^	*n*	%	*n*	%	*n*	%	
Smoking status									<0.0001
Current smoker	253	9.2	135	9.1	150	11.8	80	8.2	
Ex-smoker	990	33.7	450	30.0	304	23.6	234	25.5	
Non-smoker	1777	57.2	960	61.0	910	64.6	673	66.3	
Drinking status									0.0005
<1/year	1911	61.9	992	62.8	956	68.6	706	70.5	
1–4/month	595	20.6	296	19.5	224	17.4	146	14.6	
2–3/week	269	9.4	150	10.1	103	7.3	67	7.8	
≥4/week	248	8.2	109	7.6	84	6.7	67	7.1	
Stress status									<0.0001
Feel it very much	99	2.9	48	2.3	62	4.2	42	4.8	
Feel it somewhat	406	13.1	219	13.3	208	15.6	169	17.9	
Feel it a little	1628	55.4	860	57.6	637	47.7	433	45.1	
Feel it rarely	882	28.6	420	26.8	453	32.5	338	32.2	
Exercise									0.5960
<1/week	2310	79.3	1135	78.3	1058	81.5	751	81.3	
1–2/week	104	3.9	52	3.7	39	3.5	30	3.8	
3–4/week	145	5.4	74	5.4	58	4.6	46	5.8	
≥5/week	291	11.4	175	12.6	113	10.4	76	9.1	
Obesity status									0.2358
Underweight (BMI < 18.5 kg/m^2^)	75	2.5	54	3.6	47	3.4	30	3.7	
Normal (18.5 kg/m^2^ ≤ BMI < 23.0 kg/m^2^)	1058	36.1	508	31.9	459	33.7	350	36.3	
Overweight (23.0 kg/m^2^ ≤ BMI < 25.0 kg/m^2^)	794	25.7	403	27.4	348	25.5	247	26.3	
Obesity (BMI ≥ 25.0 kg/m^2^)	1092	35.7	590	37.1	516	37.3	354	33.7	

^(1)^ Weighted %, ^(2)^ *p*-value by chi-square test.

**Table 4:** Dietary behavior according to consumption frequency of eating alone.

Variables	Eating Together *n* = 3060		Eating Alone						*p*-Value ^(2)^
			1/Day *n* = 1574		2/Day *n* = 1394		3/Day *n* = 1009		
	*n*	% ^(1)^	*n*	%	*n*	%	*n*	%	
Snack consumption									0.0003
Yes	2794	91.63	1443	91.98	1251	89.73	876	86.72	
No	266	8.36	131	8.02	143	10.27	133	13.28	
Food security									<0.0001
Enough food—secure	1628	53.34	784	50.63	594	44.39	412	43.74	
Mildly food insecure	1330	43.24	711	45.04	681	48.31	503	48.40	
Moderately food insecure	86	2.66	71	3.73	99	6.19	69	5.69	
Severely food insecure	15	0.75	8	0.60	18	1.12	21	2.17	
Eating-out Frequency									<0.0001
≥2/day	32	1.02	24	1.46	13	1.11	10	1.09	
1/day	94	3.68	74	5.43	40	3.05	18	1.93	
5–6/week	188	6.32	129	8.22	106	7.79	27	2.62	
3–4/week	232	7.79	144	9.23	97	7.25	44	4.33	
1–2/week	765	25.53	416	26.08	352	25.39	239	23.43	
<1/week	1064	33.64	494	31.80	429	30.25	323	31.69	
seldom	685	22.02	293	17.79	357	25.17	347	34.90	

^(1)^ Weighted %, ^(2)^ *p*-value by chi-square.

**Table 5:** Food intake in their meal according to consumption frequency of eating alone.

g/Day	Eating Together *n* = 3060		Eating Alone						Unadjusted *p* for Trend ^(1)^	Adjusted *p* for Trend ^(2)^
			1/Day *n* = 1574		2/Day *n* = 1394		3/Day *n* = 1009			
	Mean	SE	Mean	SE	Mean	SE	Mean	SE		
Total food	1315.7	17.8	1298.4	19.7	1160.2	20.7	1178.0	26.5	<0.0001(−)	<0.0866(−)
Cereals and grain products	158.9	5.5	161.0	6.0	142.7	4.9	156.1	6.7	<0.0010(+)	0.0987(+)
Potatoes and starches	20.5	1.6	28.5	3.4	20.8	2.4	18.6	2.4	0.0967(−)	0.0511(−)
Sugars and sweets	3.4	0.2	3.4	0.4	2.7	0.2	2.9	0.3	0.0785(−)	0.0847(+)
Legumes and their products	22.4	1.3	28.5	2.7	22.1	1.7	30.0	3.2	0.0450(+)	0.0994(+)
Seeds and nuts	4.1	0.4	4.4	0.5	4.3	0.8	4.4	0.9	0.0724(+)	0.0882(+)
Vegetables	188.2	6.8	180.0	7.2	152.1	6.8	166.3	9.2	0.0620(−)	0.0477(−)
Mushrooms	2.8	0.3	2.4	0.4	1.2	0.2	1.5	0.4	0.0672(−)	0.0994(−)
Fruit	113.0	6.3	124.2	7.2	98.0	6.5	99.9	8.8	0.0315(−)	0.0351(−)
Meat, poultry, and their products	35.2	2.2	34.0	2.3	30.7	2.9	25.7	2.9	0.0129(−)	0.0022(−)
Eggs	10.8	0.7	11.0	0.9	8.8	0.8	9.2	1.0	0.01675(−)	0.0884(−)
Fish and shellfish	60.0	3.6	58.8	3.9	45.8	3.5	42.9	5.6	0.0453(−)	0.0729(−)
Seaweed	18.3	1.8	21.6	2.6	13.9	1.8	14.3	3.7	0.0675(−)	0.0324(−)
Milks and dairy products	30.7	2.0	34.0	2.9	38.3	3.4	39.2	3.9	0.0612(+)	0.0667(+)
Oils and fats	2.3	0.1	2.2	0.1	1.8	0.2	1.7	0.2	0.0887(−)	0.2187(+)
Beverages	62.1	4.3	72.5	5.8	52.1	5.2	35.3	4.9	0.0665(−)	0.3664(−)
Seasonings	16.6	0.7	15.7	0.7	12.9	0.6	12.9	0.9	0.0998(−)	0.0111(−)
Other food	3.1	0.5	3.8	0.8	3.8	0.8	5.0	1.3	0.0679(+)	0.0775(−)

^(1)^ *p* for trend was calculated by the SURVEYREG procedure in SAS. ^(2)^ Adjusted for gender, age, and energy intake.

**Table 6:** Nutrient intake according to consumption frequency of eating alone.

g/Day	Eating Together *n* = 3060		Eating alone						Unadjusted *p* for Trend ^(1)^	Adjusted *p* for Trend ^(2)^
			1/Day *n* = 1574		2/Day *n* = 1394		3/Day *n* = 1009			
	Mean	SE	Mean	SE	Mean	SE	Mean	SE		
Energy (kcal)	1682.7	16.7	1653.9	21.5	1517.4	20.2	1576.5	22.5	<0.0001(−)	0.5353(+)
Carbohydrates (g)	285.0	2.5	279.8	3.8	261.3	3.2	277.3	3.7	<0.0001(−)	0.0657(+)
Protein (g)	58.0	0.8	56.0	0.9	50.3	0.9	51.3	1.0	<0.0001(−)	<0.0001(−)
Fat (g)	29.3	0.7	28.3	0.6	26.0	0.7	25.6	0.8	<0.0001(−)	0.2612(+)
Calcium (mg)	464.8	7.8	457.8	9.5	426.8	9.4	434.5	10.7	0.0006(−)	0.8470(+)
Phosphorus (mg)	935.5	11.4	905.1	12.9	829.4	13.2	861.8	15.7	<0.0001(−)	0.1460(−)
Iron (mg)	11.3	0.2	11.0	0.2	10.0	0.2	10.2	0.2	<0.0001(−)	0.0550(−)
Sodium (mg)	3012.0	43.7	2815.0	51.0	2606.4	53.8	2678.5	74.1	<0.0001(−)	0.0316(−)
Potassium (mg)	2729.3	35.5	2633.1	43.4	2409.1	42.9	2441.2	51.2	<0.0001(−)	0.0012(−)
Vitamin A (μg RE)	350.2	15.0	293.5	7.2	286.6	9.8	265.1	10.1	<0.0001(−)	0.0003(−)
Carotene (μg)	2914.8	81.6	2558.2	73.4	2434.9	93.9	2255.5	95.4	<0.0001(−)	<0.0001(−)
Retinol (μg)	1.2	0.0	1.1	0.0	1.1	0.0	1.1	0.0	0.0327(−)	0.1363(−)
Thiamine (mg)	107.2	12.4	80.2	3.5	83.6	5.0	77.1	5.0	<0.0001(−)	0.8884(−)
Riboflavin (mg)	1.3	0.0	1.2	0.0	1.1	0.0	1.1	0.0	<0.0001(−)	0.1725(−)
Niacin (mg)	10.8	0.2	10.4	0.2	9.4	0.2	9.5	0.2	<0.0001(−)	0.0032(−)
Vitamin C (mg)	59.9	1.7	59.2	2.1	56.7	2.4	52.7	2.3	0.0158(−)	0.3947(−)
Energy Contribution (%, SE)										
Carbohydrates	71.4	0.3	71.6	0.3	72.3	0.3	73.2	0.4	<0.0001(+)	0.0055(+)
Protein	13.7	0.1	13.5	0.1	13.1	0.1	12.9	0.1	<0.0001(−)	<0.0001(−)
Fat	14.9	0.2	14.9	0.2	14.6	0.3	13.9	0.3	0.0100(−)	0.0233(−)

^(1)^ *p* for trend was calculated by the SURVEYREG procedure in SAS. ^(2)^ Adjusted for gender, age, and energy intake.

**Table 7:** Prevalence and mean score regarding item of Patient Health Questionnaire according to consumption frequency of eating alone.

Patient Health Questionnaire ^(1)^	Eating Together *n* = 3060		Eating Alone					
			1/Day *n* = 1574		2/Day *n* = 1394		3/Day *n* = 1009	
	Mean	SE	Mean	SE	Mean	SE	Mean	SE
1. Little interest or pleasure in doing things	0.24	0.02	0.19	0.02	0.27	0.03	0.32	0.04
2. Feeling down, depressed, or hopeless	0.18	0.02	0.24	0.03	0.29	0.03	0.35	0.05
3. Trouble falling or staying asleep, or sleeping too much	0.55	0.03	0.58	0.04	0.67	0.05	0.71	0.06
4. Feeling tired or having little energy	0.52	0.03	0.51	0.04	0.57	0.05	0.62	0.05
5. Poor appetite or overeating	0.20	0.02	0.19	0.03	0.29	0.03	0.33	0.04
6. Feeling bad about yourself—or that you are a failure or have let yourself or your family down	0.12	0.01	0.14	0.02	0.21	0.03	0.25	0.04
7. Trouble concentrating on things, such as reading the newspaper or watching television	0.11	0.01	0.13	0.02	0.14	0.02	0.16	0.03
8. Moving or speaking so slowly that other people could have noticed? Or the opposite, being so fidgety or restless that you have been moving around a lot more than usual	0.09	0.01	0.10	0.02	0.10	0.02	0.11	0.02
9. Thoughts that you would be better off dead or of hurting yourself in some way	0.12	0.02	0.09	0.02	0.19	0.03	0.20	0.03
Total score	1.56	0.09	1.80	0.14	2.06	0.15	2.40	0.23
Prevalence of depression (n, weighted %)								
Non-depression (<10) ^(2)^	1726	97.30	867	96.65	755	96.11	568	93.84
Depression (≥10) ^(3)^	64	2.70	30	3.35	38	3.89	40	6.16

^(1)^ Q. 1–9. Score (0: Not at all, 1: Several days 2: A week or higher, 3: Everyday). ^(2)^ Total score of Patient Health Questionnaire (PHQ)-9 < 10, ^(3)^ Total score of PHQ-9 ≥ 10.

**Table 8:** Relationship between daily consumption of eating alone and prevalence of depression.

	Eating Together	1/Day	2/Day	3/Day	*p* for Trend
Prevalence of depression					
Model 1	1 ^(1)^	1.410 (0.613–1.936) ^(2)^	1.437 (0.898–2.297)	2.464 (1.527–3.975)	0.0001 (+)
Model 2	1	1.093 (0.647–1.847)	1.982 (0.742–1.882)	2.152 (1.302–3.555)	0.0010 (+)
Model 3	1	1.253 (0.716–2.193)	0.942 (0.553–1.604)	1.775 (1.012–3110)	0.1286 (+)
Model 4	1	1.103 (0.613–1.985)	0.938 (0.543–1.622)	1.573 (0.871–2.838)	0.4364 (+)

^(1)^ Reference, ^(2)^ Odd ratio (95% Confidence Interval). Model 1: Unadjusted. Model 2: Adjusted for gender, age, and energy intake. Model 3: Adjusted for gender, age, family size, energy intake, exercise, alcohol consumption, stress status, smoking, and obese status. Model 4: Adjusted for gender, age, family size, energy intake, exercise, alcohol consumption, stress status, smoking, obese status, food security, snack, eating-out frequency per week, breakfast frequency per week, marital status, occupation, education level, and household income.

**Table 9:** Ratio of item of quality of life according to consumption frequency of eating alone.

Variables	Eating Together *n* = 3060		Eating Alone						*p*-Value
			1/Day *n* = 1574		2/Day *n* = 1394		3/Day *n* = 1009		
	*n*	%	*n*	%	*n*	%	*n*	%	
Mobility									<0.0001
M1. No problems	1838	60.75	966	62.23	781	58.12	475	48.30	
M2. Some/moderate problems	986	30.23	461	27.87	467	31.22	409	39.80	
M3. Extreme problems	36	0.96	14	0.66	29	1.70	24	1.99	
Non-respondence	200	8.06	133	9.24	117	8.97	100	9.91	
Self-management									0.0413
SM1. No problems	2583	83.68	1312	83.33	1124	81.05	791	79.21	
SM2. Some/moderate problems	251	7.52	117	6.52	145	9.52	110	10.32	
SM3. Extreme problems	26	0.74	11	0.84	9	0.52	7	0.56	
Non-respondence	200	8.06	134	9.31	116	8.91	100	9.91	
Daily activities									0.0002
DA1. No problems	2332	76.21	1217	77.74	996	72.72	678	68.95	
DA2. Some/moderate problems	487	14.59	211	12.42	258	16.85	216	19.78	
DA3. Extreme problems	41	1.14	13	0.59	24	1.51	13	1.32	
Non-respondence	200	8.06	133	9.24	116	8.91	101	9.95	
Pain/Inconvenience									0.0392
PI1. No problems	1849	61.09	956	60.84	780	57.93	535	53.70	
PI2. Some/moderate problems	874	26.58	412	25.73	407	27.75	308	30.28	
PI3. Extreme problems	133	4.13	72	4.12	91	5.41	63	5.95	
Non-respondence	204	8.20	134	9.31	116	8.91	102	10.06	
Anxiety/Depression									0.001
AD1. No problems	2477	80.01	1260	80.21	1065	76.11	727	73.04	
AD2. Some/moderate problems	352	10.81	168	9.88	180	13.06	159	15.46	
AD3. Extreme problems	28	1.03	13	0.66	29	1.58	20	1.54	
Non-respondence	203	8.14	133	9.24	120	9.25	102	9.96	
EQ-5D ^(1)^ index ^(2)^ (Mean, SE)	0.82 ^a^	0.008	0.821 ^a^	0.011	0.800 ^ab^	0.010	0.773 ^b^	0.012	<0.0001

^(1)^ EQ-5D: Euro Quality of Life-5 Dimension. ^(2)^ EQ-5D index = 0.05 + 0.096(M2) + 0.418(M3) + 0.046(SM2) + 0.136(SM3) + 0.051(DA2) + 0.208(DA3) + 0.037(PI2) + 0.151(PI3)+ 0.043(AD2) + 0.158(AD3) + 0.05(N3: The case given at least two points or more in more than one item). ^a,b^ different superscript letters mean significantly different among groups at the α = 0.05 level by Tukey’s multiple range comparison.

**Table 10:** Relationship between daily consumption of eating alone and quality of life.

	Eating Together	1/Day	2/Day	3/Day	*p* for Trend
**Dependent Variable: Quality of Life**					
Model 1	0 ^(1)^	0.0077 (−0.0019~0.0174) ^(2)^	−0.0152 (−0.0269~−0.0037)	−0.0347 (−0.0482~−0.0211)	<0.0001 (−)
Model 2	0	0.0074 (−0.0019~0.0166)	−0.0030 (−010140~0.0079)	−0.0205 (−0.0333~−0.0077)	0.0057 (−)
Model 3	0	0.0056 (−0.0034~0.0146)	−0.0024 (−0.0129~0.0080)	−0.0153 (−0.0271~−0.0034)	0.0273 (−)
Model 4	0	0.0066 (−0.0025~0.0156)	0.0047 (−0.0058~0.0152)	−0.0040 (−0.0173~0.0091)	0.9571 (−)

^(1)^ Reference, ^(2)^ β-coefficients (95% Confidence Interval), Model 1: Unadjusted; Model 2: Adjusted for gender, age, and energy intake; Model 3: Adjusted for gender, age, energy intake, family size, exercise, alcohol consumption, stress status, smoking, obese status, food security, snack, and eating-out frequency per week; Model 4: Adjusted for gender, age, family size, energy intake, exercise, alcohol consumption, stress status, smoking, obesity status, food security, snacking, eating-out frequency per week, marital status, occupation, education level, and household income.

## Data Availability

All data were obtained from the Korea Disease Control and Prevention Agency and are available with the permission of the Korea Disease Control and Prevention Agency. The data in this study were from the Korea National Health and Nutrition Examination Survey.
